# In vitro larvicidal efficacy of *Lantana* *camara* essential oil and its nanoemulsion and enzyme inhibition kinetics against *Anopheles* *culicifacies*

**DOI:** 10.1038/s41598-024-67148-w

**Published:** 2024-07-15

**Authors:** Shruti Sonter, Manish Kumar Dwivedi, Shringika Mishra, Prabhakar Singh, Ramesh Kumar, Sungmin Park, Byong-Hun Jeon, Prashant Kumar Singh

**Affiliations:** 1 Department of Biotechnology, Indira Gandhi National Tribal University, Amarkantak, Madhya Pradesh 484887 India; 2 R&D, Hikal Limited, Hinjawadi, Pune, Maharashtra India; 3 Sophisticated Analytical Instrumentation Facility, Department of Anatomy, All India Institute of Medical Sciences, New Delhi, India; 4 Department of Civil and Environmental Engineering, Hanyang University, 222-Wangsimni-ro, Seongdong-gu, Seoul, 04763 Republic of Korea; 5 Department of Earth Resources and Environmental Engineering, Hanyang University, 222-Wangsimni-ro, Seongdong-gu, Seoul, 04763 Republic of Korea; 6 Department of Biochemistry, University of Lucknow, Lucknow, Uttar Pradesh India

**Keywords:** Nanoparticles, Drug delivery

## Abstract

Mosquitoes are important vectors for the transmission of several infectious diseases that lead to huge morbidity and mortality. The exhaustive use of synthetic insecticides has led to widespread resistance and environmental pollution. Using essential oils and nano-emulsions as novel insecticides is a promising alternative approach for controlling vector borne diseases. In the current study, Lantana camara EO and NE were evaluated for their larvicidal and pupicidal activities against Anopheles culicifacies. The inhibitory effect of EO and NE on AChE, NSE (α/β), and GST was also evaluated and compared. GC-MS analysis of oil displayed 61 major peaks. The stable nano-emulsion with an observed hydrodynamic diameter of 147.62 nm was formed using the o/w method. The nano-emulsion exhibited good larvicidal (LC50 50.35 ppm and LC90 222.84 ppm) and pupicidal (LC50 54.82 ppm and LC90 174.58 ppm) activities. Biochemical evaluations revealed that LCEO and LCNE inhibited AChE, NSE (α/β), and GST, displaying LCNE to be a potent binder to AChE and NSE enzyme, whereas LCEO showed higher binding potency towards GST. The nano-emulsion provides us with novel opportunities to target different mosquito enzymes with improved insecticidal efficacy. Due to its natural origin, it can be further developed as a safer and more potent larvicide/insecticide capable of combating emerging insecticide resistance.

## Introduction

Malaria, filariasis, and dengue are some of the vectors borne infectious diseases that are major health problems worldwide^1^. More than a hundred species of mosquitoes have been reported for transmitting diseases in humans and vertebrates, leading to significant economic crises within the disease-endemic countries^2^. Worldwide, around 247 million cases of malaria were reported in 2021, and the estimated number of malaria deaths increased to 619,000^3^.The indispensable approach is to reduce adult mosquito population density by eliminating the larval hatching or development sites^4^. The use of larvicides, insecticides, and natural predators of mosquito larvae can pave the way for alternative methods of disease control. Targeting the mosquito larvae is one of the successful methods because the larvae are limited in movement and they cannot change their habitat to avoid the controlling activities^4,5^. Synthetic insecticides such as Dichloro Diphenyl Trichloroethane (DDT), malathion, deltamethrin, and temephoshave been extensively used as larvicides^6^. Increasing resistance to current therapies and environmental pollution, including those from synthetic larvicides or insecticides, has created a need for alternative and natural approaches that are safe and acceptable to both humans and the environment^5^ for controlling vector-borne diseases.

Amongst the emerging methods, plant-based insecticides/larvicides are gaining more attention. Plant secondary metabolites possess large chemical diversity, playing a vital role in plant defense mechanisms against diverse groups of insect pests^7^. Besides their industrial and pharmacological importance, essential oils (EOs) have also been extensively studied for their larvicidal properties. The EOs can be a potent alternative tool due to their biodegradability, minimal side effects, and enhanced efficacy^5^. Despite promising larvicidal activities, the intrinsic poor water solubility of EOs is the key technological challenge^8^, which needs to be addressed. Emerging technologies like nanoemulsions (NE) and nano-encapsulations (NEC) are extensive methods to overcome the solubility issues of EOs. Essential oil-based NEC has been recognized as a valuable product for mosquito control.

*Lantana* *camara* is an ornamental, evergreen, gregarious, erect, and hairy perennial shrub presenting a characteristic odor and growing up to 3 m tall^4^. Many pharmacological studies have been reported on the medicinal efficacy of *L.* *camara*^9^*.*The leaf extracts and EO of *L.* *camara* have exhibited potent larvicidal activities, while the flower has been reported to possess mosquito-repellent activity^4,9^. We have earlier reported some traditional methods used by tribal communities of the Amarkantak region for repelling insects^10^. In the current study, we discuss the formulation of oil-in-water (O/W) NE containing *L.* *camara* EO (LCEO) and also compare the efficacy of *L.* *camara* EO and *L.* *camara* NE (LCNE) for their potent larvicidal and pupicidal activities against *Anopheles* *culicifacies.* We further discuss the effect of LCEO and LCNE on the major enzyme targets (acetylcholine esterase, non-specific esterases, and glutathione S-transferases) of the mosquito larvae. The enzyme kinetics in the presence of the inhibitors are also discussed in the study.

## Results

### Plant collection and extraction

*L.* *camara* is one such plant that is being used by the tribal communities and was used in the current study for extraction of the EO. Yellow-colored LCEO with a characteristic aroma was steam distilled with a yield of 0.3 mL/100 g fresh leaves.

### Characterization of *Lantana* camara Oil

Characterization of the LCEO using GC and GC–MS displayed 61 compounds, of which 17 major compounds were identified (Figure S1 and S2). Eudesm-7(11)-e-4-ol (14.72%), aromadendrene (12.90%), α-pinene (10.2%), camphor (7.8%), γ-terpinene (7.53%), p-cymene (7.43%), β-selinene (5.11%), (Z)-β-ocimene (4.1%), (E)-β-ocimene (3.6%), azulene (3.95%), veridiflorol (2.93%), β- humulene (2.35%), γ-cadinene (1.93%), linalool (1.7%), trans-caryophellene (1.3%), α-thujene (1.3%) and germacrene D (1.29%) were the major compounds.The remaining 35 compounds contributed in minor quantities (1.1–0.05%), while the other 9 compounds were present in traces (< 0.05%) (Table 1). Compounds such as Eudesm-7(11)-e-4-ol, aromadendrene, camphor, γ-terpinene, p-cymene, and β-selinene were major constituents of LCEO, requiring further fractionation, purification, and identifying the probable mode of mechanism for larvicidal activity.

**Table 1:** Chemical composition of *L.* *camara* essential oil (GC–MS).

SI No.	Name of compounds	RI_Cal_	RI_Lit_	Concentration (% peak area)
1.	α-Thujene	911	924	1.30
2.	α-pinene	933.2	932	10.22
3.	Sabinine	980.4	975	0.23
4.	β-myrcene	995.2	988	Tr
5.	p-Cymene	1026	1020	7.43
6.	1,8-cineole	1040	1026	0.25
7.	(E)-β-Ocimene	1046	1044	3.61
8.	(Z)-β-Ocimene	1047	1032	4.16
9.	Υ-Terpinene	1062	1054	7.52
10.	cis-sabinine hydrate	1065	1065	0.82
11.	trans-sabinine hydrate	1092	1098	0.15
12.	Linalool	1107	1095	1.71
13.	Norborneol acetate (exo-2-)	1125	1125	0.14
14.	Camphor	1149	1141	2.23
15.	Borneol	1156	1165	0.11
16.	Camphor	1157	1141	5.67
17.	Terpinen-4-ol	1172	1174	0.45
18.	α-Terpineol	1191	1186	0.80
19.	cymene-9-ol (p)	1207	1204	0.06
20.	Linalyl acetate	1227	1231	0.65
21.	cumin aldehyde	1230	1238	Tr
22.	Linalool acetate	1254	1254	0.19
23.	Bornyl acetate	1272	1287	Tr
24.	m-Thymol	1280	1290	0.12
25.	Azulene	1308	1298	3.95
26.	Verbanol acetate	1322	1322	Tr
27.	α- Cubebene	1345	1345	Tr
28.	α-Ylangen	1349	1373	0.14
29.	β- Elemene	1389	1389	0.29
30.	β-Caryophyllene	1404	1401	0.11
31.	trans-caryophyllene	1415	1419	1.37
32.	β-Humulene	1436	1436	2.35
33.	Aromandendrene	1440	1441	12.95
34.	α- Humulene	1449	1452	0.58
35.	trans-Muurola-3,5-diene	1453	1453	Tr
36.	trans-b-Farnesene	1456	1457	0.18
37.	Aristolochene (4,5-di-epi)	1474	1473	0.12
38.	Υ- murolene	1477	1478	0.11
39.	Germacrene D	1481	1484	1.29
40.	β- Selinene	1489	1489	5.1
41.	α- Bisabolene	1510	1507	0.10
42.	Germacrene A	1515	1509	0.24
43.	Υ-Cadinene	1522	1513	1.93
44.	Italicene ether	1543	1537	0.52
45.	cis-Muurol-5-en-4-beta-ol	1549	1551	0.21
46.	Germacrene B	1564	1561	Tr
47.	Spathulenol	1576	1578	Tr
48.	Caryophyllene oxide	1586	1583	0.08
49.	Veridiflorol	1590	1592	2.3
50.	Guaiol	1604	1600	0.35
51.	β-Himachalene oxide	1613	1618	0.16
52.	Epi-bicyclosesquiphellandrene	1625	1625	0.35
53.	1-epi-Cubenol	1628	1628	0.29
54.	c-Eudesmol	1636	1632	0.32
55.	epi-a-Muurolol	1651	1654	Tr
56.	Geranyl valerate	1658	1656	0.5
57.	–	1667	Nd	Tr
58.	Helifolenol B	1678	1678	0.3
59.	Eudesm-7(1l)-en-4-ol	1705	1700	14.7
60.	–	1730	Nd	0.6
61.	–	1754	Nd	0.18
62.	Essential oil yield %(v/w)^a^	–	–	0.30
63.	Total compounds identified	58		

RI_cal_, Retention indices calculated; RI_Lit_, Retention indices from literature (Adams, 2007); Tr, trace amount; Nd, not identified. ^a^Yield of isolated oils expressed as mL of essential oil/100 g of fresh leaves.

### UV–Vis spectrometric study of nanoemulsion

**Figure 1:**
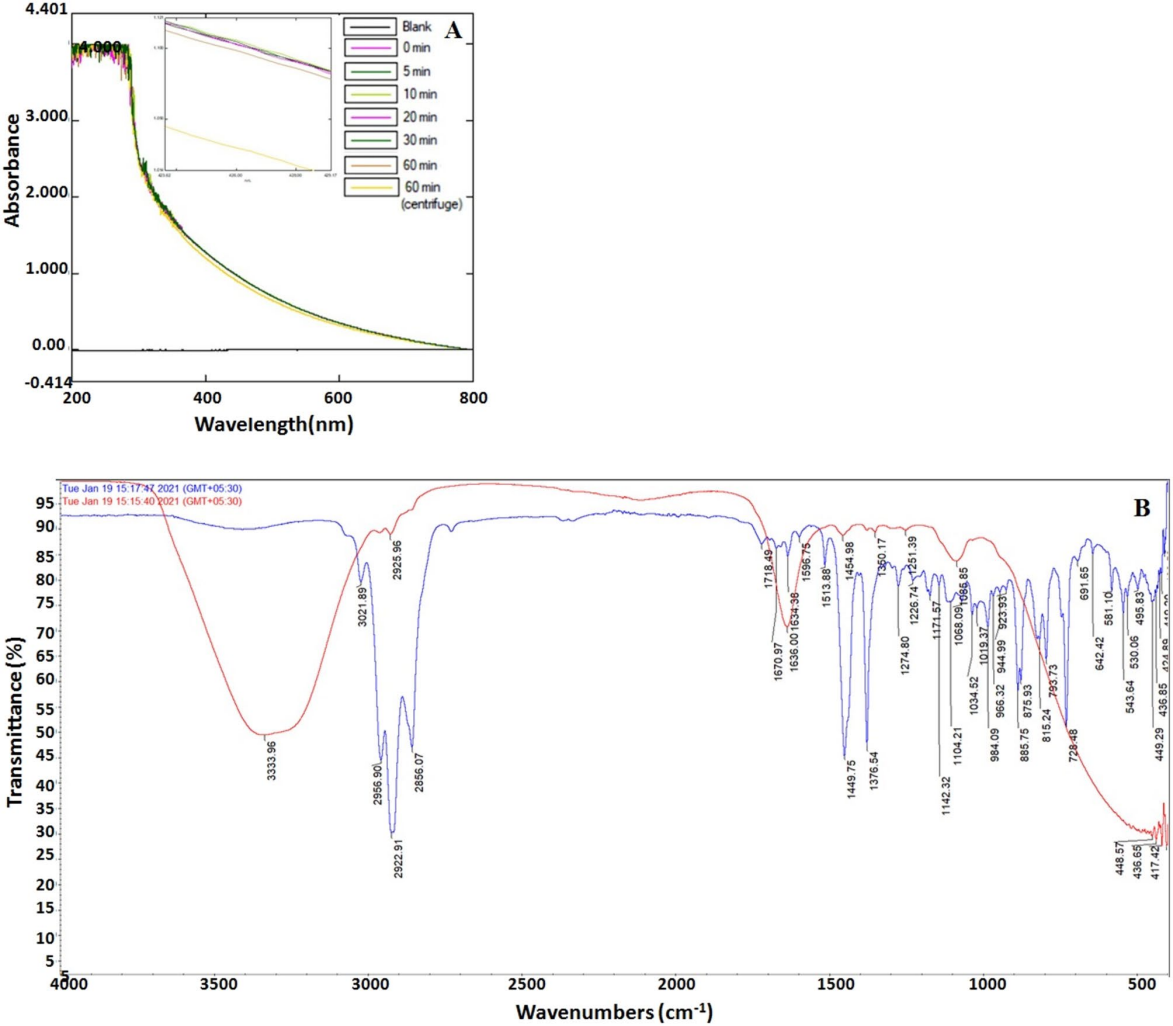
(**A**) UV-Spectroscopic analysis of nanoemulsion prepared at different time (5, 10, 20, 30 and 60 min) intervals, (**B**) FTIR vibration spectra of *L.* *camara* oil (blue) and FTIR vibration spectra of LCNE (Red).

UV–Vis spectrophotometry has usually been utilized for the measurement of turbidity and evaluation of the stability of the emulsions^11^. The broad spectrum of LCNE was observed at 400 nm (Fig. 1A). The time-dependent UV–Vis spectrum displayed neither new peak nor there were major peak shifts. There was a slight change in the spectrum when the LCNE was centrifuged and analyzed for any changes, confirming the stable nature of the emulsion.

### FTIR spectrometry study of *L. camara* essential oil and nanoemulsion

The FTIR spectrum is a potent tool for identifying the functional groups present in the desired sample based on the peak value between the regions of 400–4000 cm^−1^. It is also a fast and non-destructive method for fingerprinting any sample. The FTIR spectrum of LCEO (Fig. 1B) displayed an absorption peak at 3072.7 cm^−1^ due to C–H– stretch, confirming the sp^2^ hybridization, peak values 2954.8, 2924, and 2867.3 cm^-1^ were due to the presence of CH– symmetric stretching of saturated (sp^3^) compound. The absorption at 2725.2 cm^−1^ depicts the presence of C–H stretching for the aldehyde group. The notable peak at 1642–1603 cm^−1^ showed C=C variable of the alkene group, 1514 cm^−1^, due to aromatic ring deformations. A peak at 1446.5 cm^−1^ showed C–H (bend/scissoring), 1381.9 cm^−1^ due to C–H rock of methyl stretching, and 1302.8 cm^−1^ showed C–O stretching of aromatic esters. The peak value of 1276.47, 1254.53, 1224.72, 1181.39, 1165.31, 1104.54, and 1055.36 cm^−1^ can be associated with C–O stretching of alkyl/aryl ether, ester, tertiary, secondary and primary alcohols respectively. The absorption peaks at 980.22 and 969.64 cm^−1^ are because of strong C=C bending of mono and di-substitution, whereas the peaks at 885.16–814.38 cm^−1^ confirm the C–H bending of 1,2,3 tri-substitute and the 786.35–720.40 cm^−1^ which indicated the presence of –C–H– rocking of the long chain. The FTIR spectrum of LCNE is shown in Fig. 1C.

### Particle size and stability analysis of the nanoemulsion

The LCNE obtained by the O/W method yielded a bright white emulsion upon the addition of water to the mixture of Tween-20 and oil. The droplet size distribution and PDI of LCNE are significant in estimating physical properties and particle distribution. The particle sizes of the LCNE were 184, 147, 150, 151, and 161 nm for time (5, 10, 20, 30, and 60 min) (Figure S3 and Table 2). At 5 min of stirring, the size was comparatively larger, decreased from 10 min onwards, and remained more or less static. The nanometric size of the emulsion did not alter even at 100 times dilution with water, proving that the emulsion was well-suited to aqueous fluids. The increased absorption capacity of nanoemulsion may be attributed to the large specific area available due to the small particle size of nanoemulsion droplets. Investigation on LCNE stability displayed no phase separation by centrifugation, and the thermodynamic cycles neither altered the physical appearance nor the color of the LCNE. There was minimal change in the UV absorbance before and after centrifugation, which confirmed the stability of the LCNE (Fig. 1A). Table 2 shows the hydrodynamic diameter, PDI, and zeta potential (ZP) of time-dependent LCNE formulation. The observed zeta potential (− 26.5) at 10 min of stirring indicates the formation of stable LCNE that was further used in all the assays. The observed negative charge of the LCNE (Figure S4) might be due to the anionic groups of the fatty acids and glycols present in the surfactant Tween-20 used in the formulation of LCNE.

**Table 2:** Particle size, polydispersity index and zeta potential of *L. camara *nanoemulsion.

Sample code	Particle size (Hydrodynamic diameter) in nm	Polydispersity index (PDI) in %	Zeta potential in mV Mean ± SD
LCNE-05	184.87	3.2	− 18.7
LCNE-10	147.62	15.1	− 26.5
LCNE-20	150.31	14.1	− 05.5
LCNE-30	151.50	18.4	− 04.5
LCNE-60	161.14	21.5	− 12.5

LCNE, *Lantana* *camara* nanoemulsion, 05, 10, 20, 30 and 60 is time in mins.

### Transmission electron microscopy

**Figure 2:**
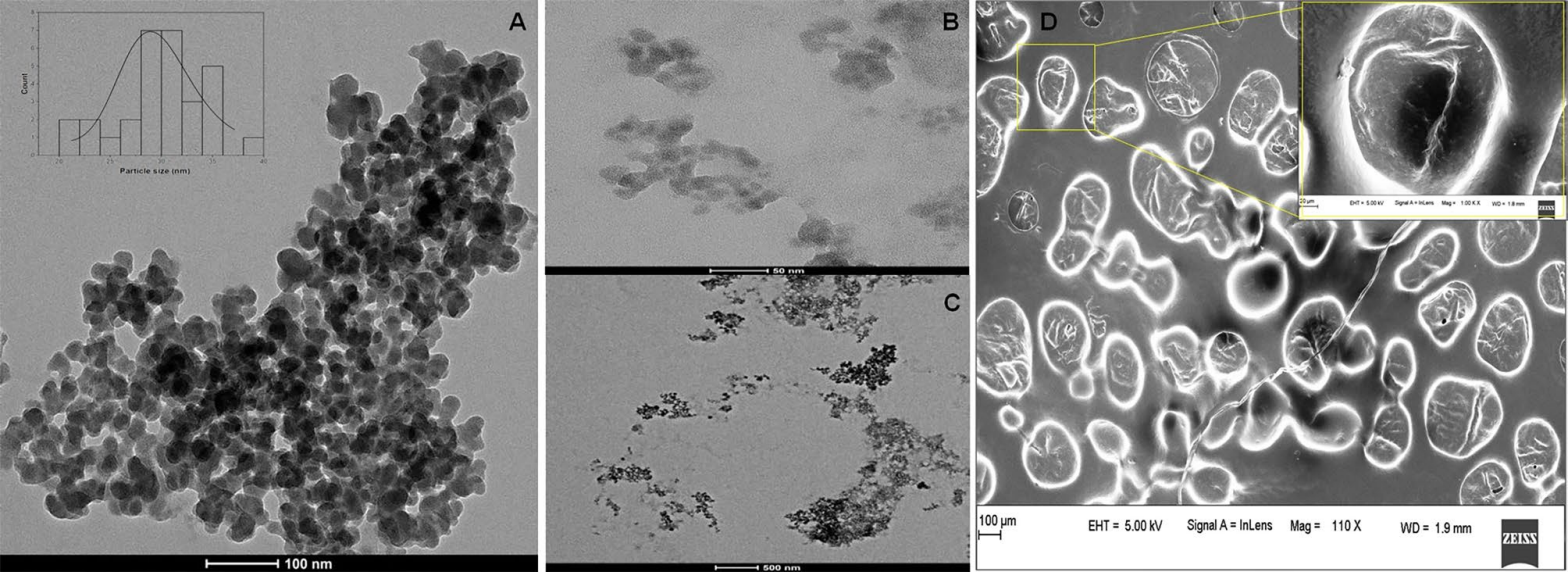
(**A**) TEM analysis of LCNE at 100 nm scale (**B**) TEM analysis of LCNE at 50 nm scale (**C**) TEM analysis of LCNE at 500 nm scale (D) SEM analysis of LCNE at 110× magnification.

LCNE particle shape and size wereanalyzed by TEM, as demonstrated in Fig. 2A. The results revealed that the LCNE are spherical and monodisperse. The quantitative analysis performed using the TEM image revealed the average particle size of LCNE to be around 29.7 ± 4.4 nm, as determined by a histogram fitted by the Lorentzian function.

### Scanning electron microscopy

The morphology of LCNE observed by SEM has been presented in Fig. 2D.The LCNE were distributed uniformly and were almost spherical. The solidifying/drying process did not change the structure of aggregates. At the highest magnitude used, dried LCNE appeared to be single spherical units, some linked to each other to form dimeric units. The external surface of each unit is almost regular and smooth, displaying that the Tween-20 creates a continuous film surrounding the essential oil droplets.

### Larvicidal and pupicidal activity of LCEO and LCNE

**Figure 3:**
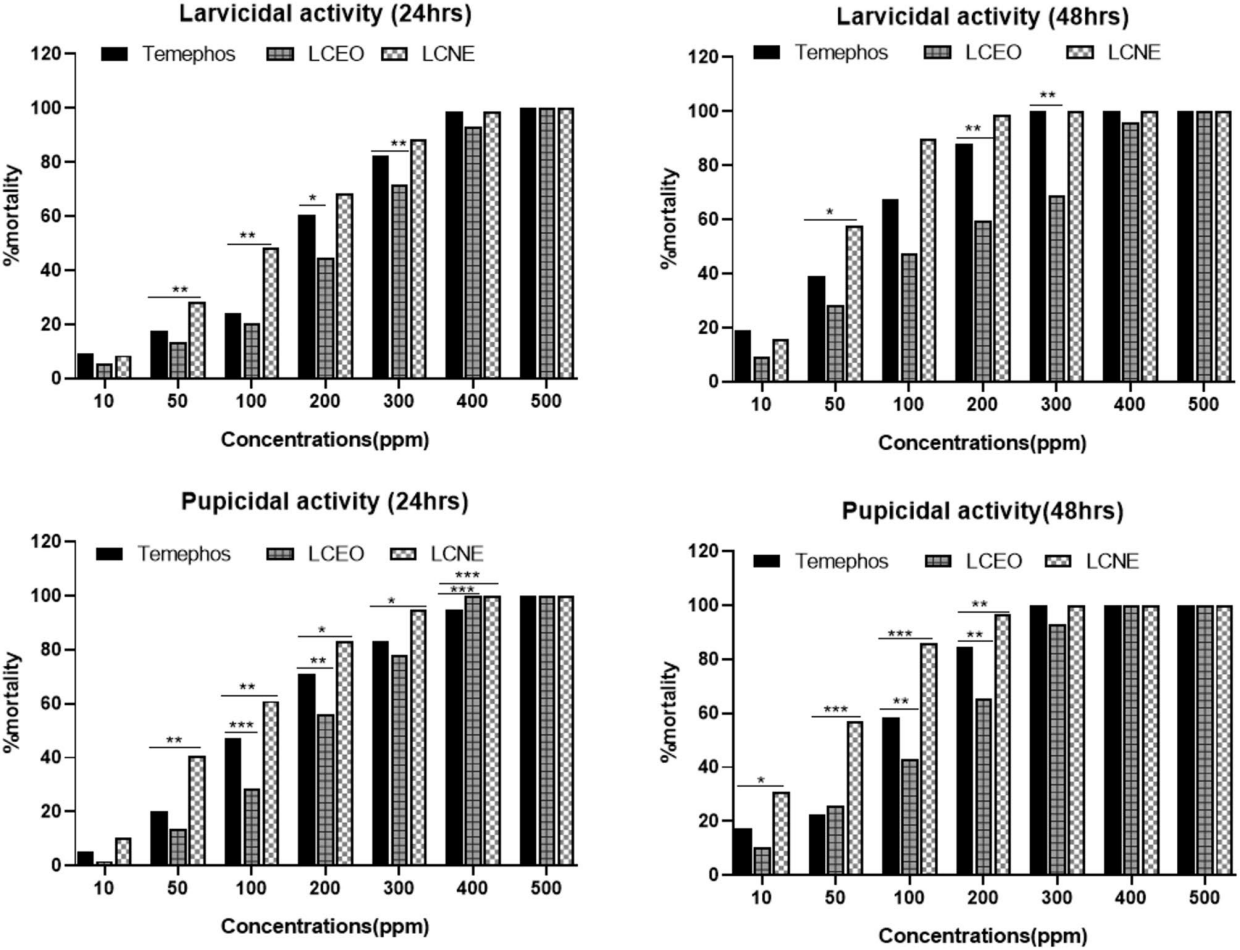
(**A**) Larvicidal activity of fourth instar larvae at 24 and 48 h of exposure with Temephos, LCEO and LCNE. Graph plotted against %mortality and concentration in ppm. LCEO: *L.* *camara* essential oil, LCNE: *L.* *camara* nanoemulsion. (**B**). Pupicidal activity at 24 and 48 h of exposure with Temephos, LCEO and LCNE. Graph plotted against %mortality and concentration in ppm. LCEO: *L.* *camara* essential oil, LCNE: *L.* *camara* nanoemulsion. *p* value < 0.033 was considered significant and marked as *, *p* < 0.002 as highly significant and marked as **, *p* < 0.001 was very highly significant and marked as ***.

The *L. camara* essential oil (LCEO) and *L. camara* nanoemulsion (LCNE) were evaluated for larvicidal and pupicidal activities. The results strongly support the potential of essential oils and NE’s for the development of plant-based insecticides with superior activities as compared to the synthetic insecticide temephos. Both LCEO and LCNE exhibited potent larvicidal activities comparable totemephos (Fig. 3A). In larvicidal assays, 100% mortality was observed after 30 min in all three groups at 500 ppm. However, the mortality rates for LCNE were higher than the oil alone or temephos at all time points and concentrations (Table S1). The LC_50_ values for temephos, LCEO, and LCNE are 86.89, 111.17, and 50.35 ppm while LC_90_ values are 357.27, 450.81, and 222.84 ppm, respectively, after exposure of 24 h (Table 3). Both LCEO and LCNE also displayed potent pupicidal activities at 24 and 48 h of incubation against *An.* *culicifacies* pupae (Fig. 3B) that were higher at all time points than the standard temephos (Table S2).

**Table 3:** LC_50_ and LC_90_ values from larvicidal and pupicidal assays after 24 hrs of exposure.

Sample	LC_50_(ppm)	LC_90_(ppm)	Slope ± SD	Intercept	R^2^
Temephos	86.89	357.273	2.18 ± 0.67	0.95	0.66
LCEO	111.17	450.81	1.98 ± 0.59	0.88	0.70
LCNE	50.35	222.84	1.98 ± 0.47	1.62	0.78

Sample	LC50(ppm)	LC_90_(ppm)	Slope ± SD	Intercept	R^2^
Temephos	76.2	266.5	2.35 ± 0.45	0.56	0.84
LCEO	80.53	255.85	2.74 ± 0.69	-0.23	0.76
LCNE	54.82	174.58	2.77 ± 0.37	0.18	0.92

LCEO and LCNE displayed a 100% mortality rate at 400 ppm, while temephos led to 100% mortality at 500 ppm. After 24 h of exposure to the test substances and temephos, the calculated LC_50_ values were 76.2, 80.53, and 54.82 ppm, whereas LC_90_ values were 266.5, 255.85 and 174.58 ppm for temephos, LCEO and LCNE, respectively (Table 3). In the regression analysis of the larvicidal assay, the observed R^2^ values were higher for LCNE (0.78) LCEO (0.70) as compared to the temephos (0.66). However, in the pupicidal assay, the R^2^ values for LCNE (0.92) were higher as compared to that obtained for temephos (0.84), but LCEO (0.76) displayed little lower values (Table 3). The current results prove that the LCNE is more efficient in causing larval/pupa mortality than the LCEO.

### Biochemical analysis

In our assays, LCEO and LCNE exhibited promising inhibitory activities against key mosquito enzymes (Table 4). Further, the enzyme kinetics were carried out with varying substrate concentrations, and the inhibitor was kept constant to identify the type of inhibition. Table 5 shows the maximum enzyme velocity (Vmax), Michaelis constant (Km), and catalytic constant (Kcat) for all the enzymatic activities.

**Table 4:** Dose response studies of inhibitors on enzymes.

Sample	LCEO			LCNE		
	Log IC_50_	IC_50_ ppm	K_i_ ppm	Log IC_50_	IC_50_ ppm	K_i_ ppm
Acetylcholine esterase ````(AChE)	1.65 ± 0.025 (1.59–1.70)*	44.83*** (39.68–50.68)^#^	33.97	1.59 ± 0.023 (1.54–1.64)^#^	39.36*** (35.15–44.09)*	74.65
Α-Naphthyl acetate esterase (A-NAE)	1.685 ± 0.087 (1.498–1.872)*	48.42* (31.49–74.54)^#^	28.28	1.561 ± 0.066 (1.414–1.69)^#^	36.37** (25.94–49.75)^#^	39.82
Β-Naphthyl acetate esterase (B-NAE)	1.622 ± 0.061 (1.494–1.750)^#^	41.85*** (31.16–56.21)^#^	10.19	1.405 ± 0.115 (1.106–1.64)^#^	25.40*** (12.76–44.44)^#^	40.73
Glutathione S-Transferases (GST)	1.566 ± 0.04 (1.468–1.662)^#^	36.79*** (29.37–45.97)^#^	13.84	1.548 ± 0.03 (1.465–1.62)^#^	35.28*** (29.20–42.34)^#^	12.19

^#^indicate the 95% confidence interval (CI), *p* value < 0.033 was considered significant and marked as *, *p* < 0.002 as highly significant and marked as **, *p* < 0.001 was very highly significant and marked as ***.

**Table 5:** Enzyme kinetics parameters of Acetylcholine Esterase, α and β *naphthyl* *acetate* esterase and Glutathione S-transferases.

`Enzyme	Inhibitors	V_max_(Cl)	K_m_(Cl)	V_max_/K_m_	K_cat_(s^-1^)	K_cat_/K_m_
AChE	Control	179.3 (128.4–257.6)	3.136 (0.67–11.64)	57.17	3.002	0.957
	LCEO	95.80 (83.38–110.9)	2.891 (1.50–5.29)	33.13	1.604	0.554
	LCNE	67.77 (59.12–78.20)	2.616 (1.35–4.82)	25.90	1.135	0.433
Α-NAE	Control	491.1 (322.7–832.5)	4.147 (0.35–21.89)	118.4	7.213	1.742
	LCEO	437.6 (375.0–514.1)	6.248 (3.402–10.52)	70.03	6.427	1.028
	LCNE	418.9 (370.5–475.8)	5.519 (3.381–8.528)	75.90	6.152	1.114
Β-NAE	Control	135.2 (97–207.2)	17.26 (6.934–42.23)	7.83	1.985	0.115
	LCEO	81.49 (58,024–115.0)	5.902 (0.97–16.96)	13.80	1.197	0.202
	LCNE	67.60 (55.05–92.27)	2.306 (0.557–8.94)	29.31	0.993	0.430
GST	Control	214.5 (165.9–219)	14.89 (7.427–29.86)	14.40	3.440	0.231
	LCEO	120.2 (92.38–160.9)	8.778 (3.306–19.49)	13.69	1.927	0.219
	LCNE	104.9 (86.22–130.3)	4.012 (1.408–9.035)	26.14	1.683	0.419

#### Acetylcholine esterase

**Figure 4:**
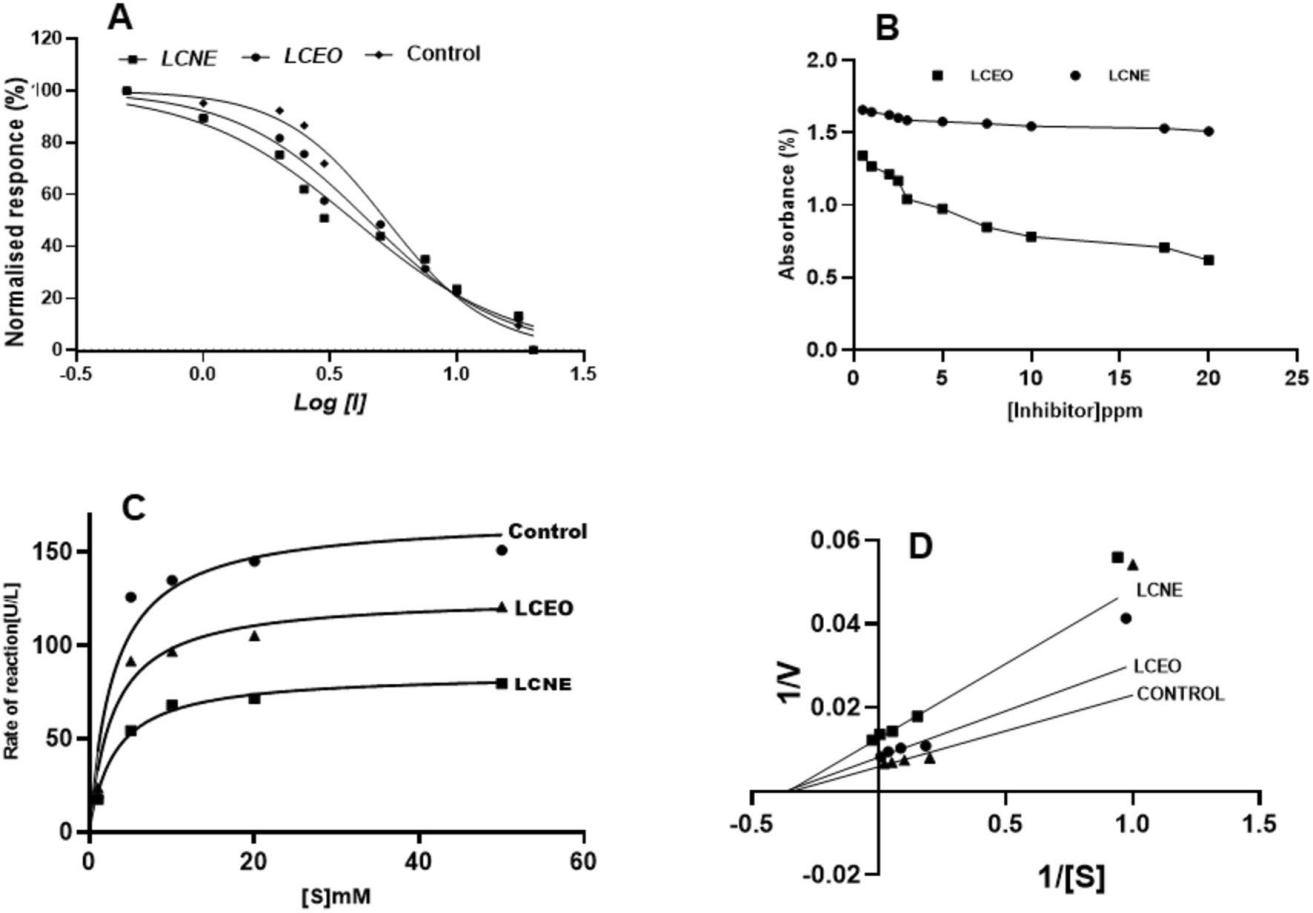
Acetylcholine esterase inhibition activity in the presence of LCEO, LCNE and absence of inhibitor (control). (**A**). Dose-dependent inhibition of Acetylcholine esterase. (**B**). Inhibitory constant (Ki), (**C**). Michaelis–Menten Graph. (**D**). Lineweaver Burk plot.

The AChE inhibition observed in LCNE was slightly more than that of LCEO. The IC_50_ values of LCEO and LCNE were 44.83 and 39.36 ppm, and Ki values were 33.97 and 74.65 ppm, respectively (Fig. 4A and B). The observed inhibitory potency of LCNE is almost double that of LCEO. Figure 4C and D show the competitive inhibition of AChE by LCEO and uncompetitive inhibition by LCNE compared to the control. The Vmax values in control, LCEO, and LCNE were 179.3, 126.2, and 85.05 UL/min, respectively.

#### Non-specific esterase

##### α-Naphthyl acetate esterase (α-NAE)

**Figure 5:**
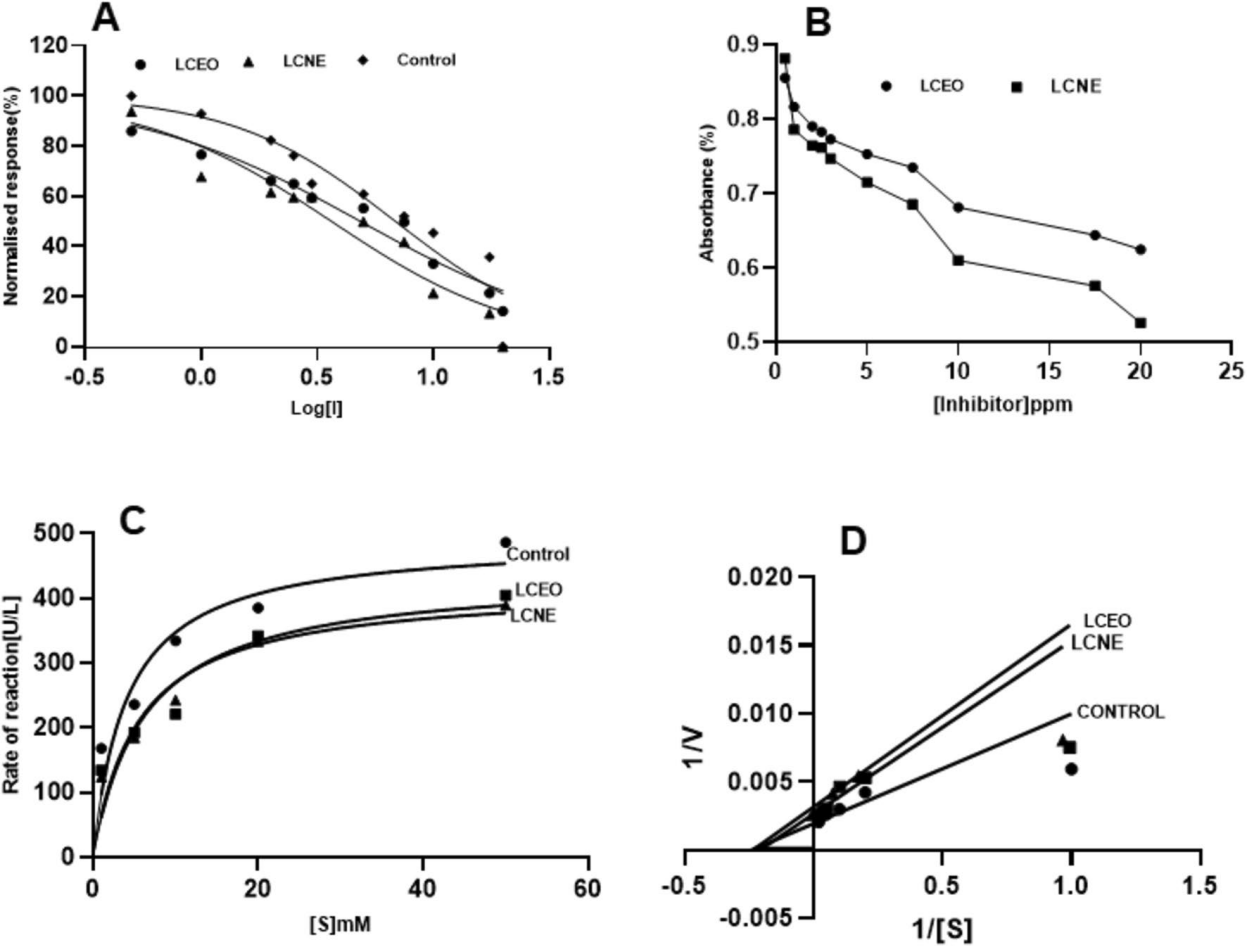
*α-naphthyl* *acetate* *esterase* inhibition activity in the presence of LCEO, LCNE and absence of inhibitor (control). (**A**). Dose dependent inhibition of α-naphthyl acetate esterase. (**B**). Inhibitory constant (Ki) (**C**). Michaelis–Menten Graph (**D**). Lineweaver Burk plot.

The observed IC_50_ values of LCEO and LCNE are 42.42 and 36.37 ppm. In contrast, the Ki values were 28.28 and 39.82 ppm, respectively, suggesting a minimal difference in the nanoemulsion inhibitory potency (Ki) compared to the LCEO (Fig. 5A and B). The observed Vmax of control, LCEO, and LCNE were 491.1, 437.6, and 418.9UL/min, respectively (Fig. 5C). LCEO and LCNE observed noncompetitive inhibition of α-NSE as compared with the control (Fig. 5D).

##### β-Naphthyl acetate esterase (β-NAE)

**Figure 6:**
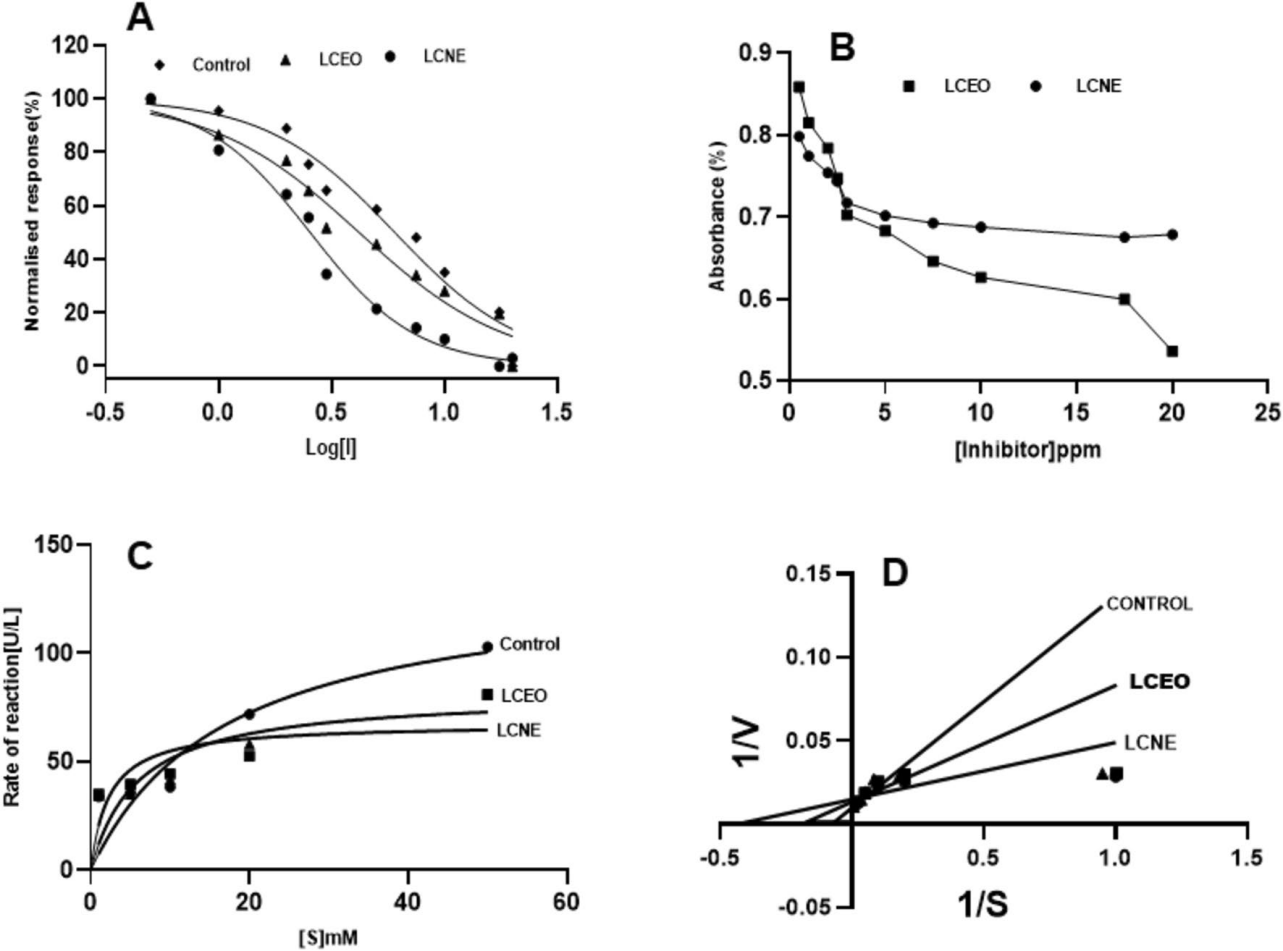
β*-naphthyl* *acetate* *esterase* inhibition activity in the presence of LCEO, LCNE and absence of inhibitor (control). (**A**). Dose dependent inhibition of β-naphthyl acetate esterase. **(B**). Inhibitory constant (Ki). (**C**). Michaelis–Menten Graph. (**D**) Lineweaver Burk plot.

The observed IC_50_ values of LCEO and LCNE are 41.85 and 25.40 ppm, respectively. Almost four times more inhibitory potency was exhibited by the oil (Ki:10.1973 ppm) than the nanoemulsion (Ki: 40.73 ppm) (Fig. 6A and B). The Vmax of control, LCEO, and LCNE were 135.2, 81.49, and 67.60UL/min, respectively (Fig. 6C). The competitive inhibition of *β-NAE* by LCEO and LCNE was observed compared to the control (Fig. 6D).

#### Glutathione S-transferases

**Figure 7:**
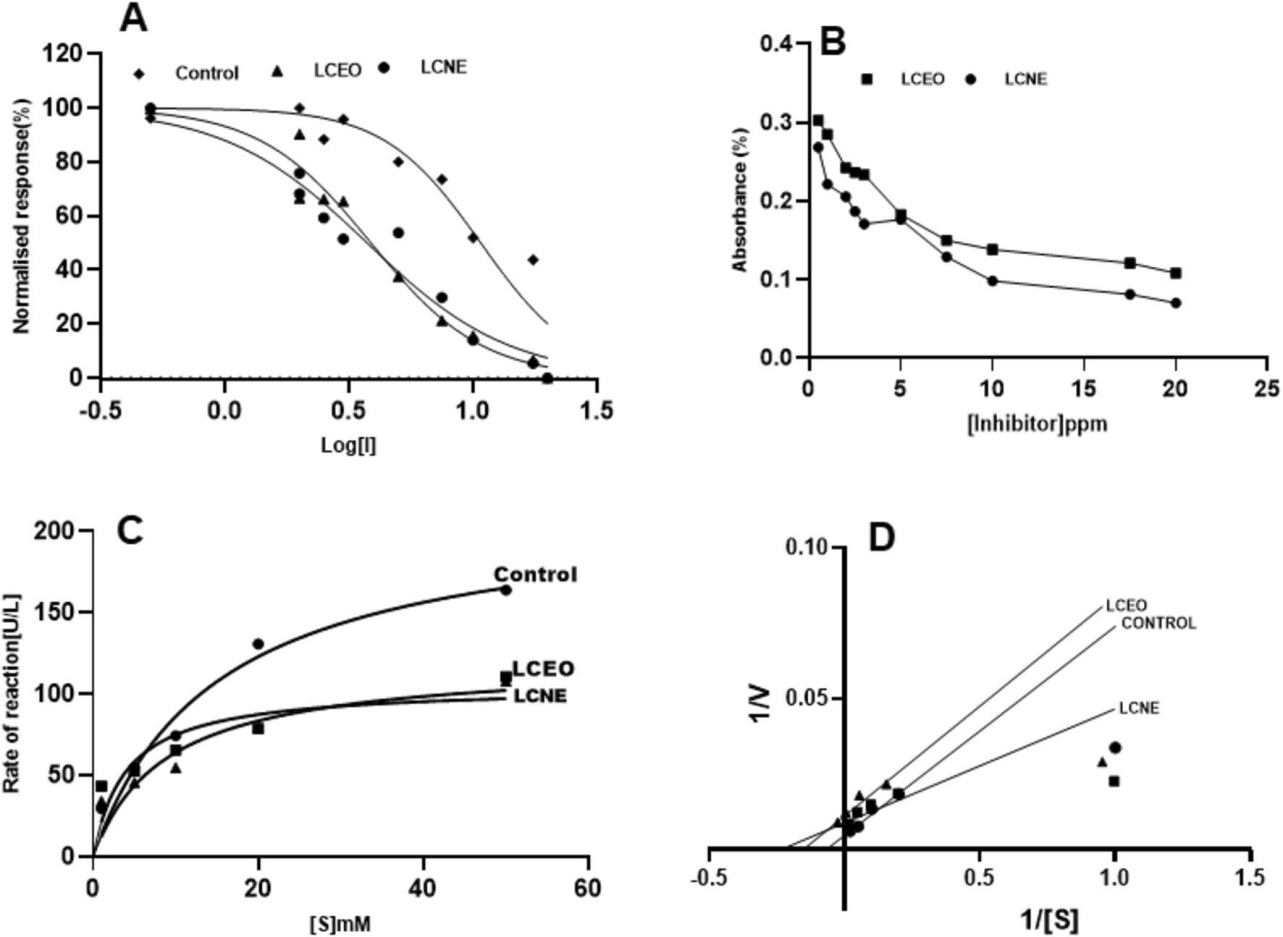
Glutathione S-Transferases inhibition activity in the presence of LCEO, LCNE and Absence of inhibitor (control). (**A**). Dose dependent inhibition of Glutathione S-Transferases. (**B**). Inhibitory constant (Ki). (**C**). Michaelis–Menten Graph. (**D**). Lineweaver Burk plot.

Similar enzymatic inhibition was observed by both LCEO and LCNE as observed by the IC_50_ values of LCEO (36.79 ppm) and LCNE (35.28 ppm). The inhibitory potency (Ki) values for LCEO and LCNE were 13.84 and 12.19 µM^-1^ mL^-1^, respectively, demonstrating slightly higher efficiency in LCEO (Fig. 7A and B). The calculated Vmax values were 214.5, 120.2, and 104.9UL/min for control, LCEO, and LCNE, respectively (Fig. 7C). A mixed type of GST inhibition by LCEO and LCNE was observed when compared to the control (Fig. 7D).

## Discussion

Malaria accounted for an estimated 229 million cases worldwide in 2019^3^. Nineteen sub-Saharan African countries and India carry almost 85% of the global malaria burden. Malaria is mostly transmitted through the bites of female *Anopheles* mosquito spp. *An.* *culicifacies* is a major malarial vector that contributes to around 65% of malaria cases in India. Vector control is the main way to prevent and reduce malaria transmission where DDT, malathion, deltamethrin, cyfluthrin, temephos, alpha-cypermethrin, and lambda-cyhalothrin are the commonly used insecticides. Resistance to DDT in *An.* *culicifacies* has been reported extensively from all over the country and against malathion from the states of Maharashtra, Gujarat, Tamil Nadu, and Uttar Pradesh. The survey by Mishra et al*.* reported that nine tribal dominant districts of Madhya Pradesh harbor insecticide-resistant strains of *An.* *culicifacies*^12^. WHO Global Report on Insecticide Resistance in Malaria Vectors: 2010–2016 reported that resistance to four commonly used insecticide classes such as pyrethroids, organochlorines, carbamates, and organophosphates is widespread in all major malaria vectors across the WHO regions. *L.* *camara* is one such plant that is being used by the tribal communities and was used in the current study for extraction of the EO.

Previous studies have displayed the potency of pure compounds in the EO for their larvicidal activities. The compounds such as germacrene D, α-pinene, and caryophyllene exhibited potent larvicidal activities^5^. Due to the presence of exocyclic double bonds, β-caryophyllene is more efficient than α-pinene in which endocyclic double bonds are present^13,14^. In our results, these compounds were observed as the major constituents of LCEO. Other compounds like sabinene, cubebene, humulene, and limonene were also present in the LCEO, requiring further fractionation, purification, and identification of the compounds with larvicidal activities. Due to its diverse chemical nature, EO has been exploited mainly to evaluate bactericidal, virucidal, fungicidal, and insecticidal activities. The practical use of EO is often limited due to several disadvantages arising out of handling or during storage. The EO undergoes chemical conversion, isomerization, polymerization rearrangement and/or degradation due to heat, humidity, light, or oxidation, leading to an overall loss of effectiveness^15,16^. EOs are known to be comprised of several dozen active substances with 2–3 main compounds in high abundance and other compounds in traces. Identification of the major active compound from the EO is cumbersome due to its complex nature that varies depending on the harvest season and the method of extraction used. The attribution of the mode of action of a particular constituent of the EO is difficult as the biotic efficacy of EOs is mainly because of the combination and composition of these compounds present within^17,18^. Compounds with similar structures are known to display additive rather than synergistic effects while antagonistic effects are attributed to the interactions amongst non-oxygenated and oxygenated monoterpene hydrocarbons ^19^. Few studies have been performed to study the synergistic nature of the EO constituents. Pavela carried out the acute toxicity and the binary mixture abilities on of 3^rd^ instar larvae of an important polyphagous pest, *S.* *littoralis*. In total, 435 combinations of the constituents were assessed, of which 150 combinations showed a significant antagonistic effect and 135 combinations exhibited a significant synergic effect on the mortality. Out of tested combinations the binary mixture of camphor/borneol, showed the highest synergic effect with ratio 1:2 respectively. It was reported that increased synergetic effects were observed in the compounds having methyl and methoxy functional groups^20^. However, the presence of carbonyl group and the absence of methyl group in certain compounds reduced the synergetic efficacy. Pavela et al. also reported that the binary mixture of Alpha-pinene/linalool and Alpha-pinene/γ-terpinene shows the synergetic effect whereas the combination of Alpha-pinene/camphor and Alpha-pinene/p-cymene does not enhance the activity. The combination of p-cymene/linalool, p-cymene/ γ-terpinene and p-cymene/camphor displayed synergetic effect. The γ-terpinene/camphor and γ-terpinene/linalool also exhibited their synergetic effectiveness against tested organism^21^. In our study, compounds such as camphor, γ-terpinene, p-cymene, alpha-pinene and linalool were major constituents of LCEO confirming that there might be synergetic effectiveness of these compounds against the tested mosquito larvae. Additionally, EOs display poor physicochemical properties such as high volatility, water insolubility, and quick half-life, making the use of EO difficult in most cases despite the significant biological activities. Oil-based NEs, due to their unique and versatile properties and applications have emerged as the delivery systems with increased bioavailability and effectiveness of the EO^22^.

In the spectra, it was observed that some peaks were masked in the LCEO spectra. The broad peak at 3331.57 and 1636.03 cm^−1^ represents the hydrophilic interaction (O–H stretch) it co-relates with IR of water^23^, 2926.2 cm^−1^ peak due to C–H stretching of alkene while 1350.25 cm^−1^ due to O–H bending. The peaks at 1456.39 and 1088.47 cm^−1^ are due to C–H bending of alkene and C–O stretching from primary alcohol, respectively. These (2926.22–1088.47 cm^−1^) peaks considerably match the IR of Tween 20^24^. The results confirm the proper encapsulation of the drug with surfactant and validate the formation of NE^25^.

NE droplet size, polydispersity index (PDI), span, and zeta potential can be evaluated by using a particle size analyzer. The PDI is a measure of the broadness of the size distribution and shows the quality/ heterogeneity of the dispersion^26^.The polydispersity could be specified by uniformity and span. The uniformity shows how symmetrically the particles are distributed around the median point, whereas the span provides the width of the distribution^27^. Zeta potential indicates the charge on the particles and also explains the degree of repulsion between “similarly charged particles” that are adjacent to each other. The observed values of zeta potential show the interactive forces between the particles at the surface of the emulsion and the stability of the NE. The zeta potential higher than ± 30 mV indicates electrically stable NE^28^. The variation in the zeta potential depends upon several factors, such as the particle source, use of different surfactants, electrolyte concentration, particle morphology and size, pH of the solution, and hydration state^29^. The results of PDI and TEM correlate with each other; the PDI %, less than 20 is monodisperse^30^.The presence of larger and smaller droplets in minor proportions might be due to undiluted samples^31,32^.

LC_50_ and LC_90_ are also considered leading parameters of the efficacy of any drug/ inhibitor for further characterization. Most of the studies have reported the LC_50_ and LC_90_ but have not undertaken further studies to understand the mechanism of action of the EO or its components. It is known that the LC_50_ is sufficient to get maximum mortality, but it should also be effective for a longer duration and against different mosquito species. The concentration required to obtain maximum mortality also depends on the insect stage, temperature, penetration of substance into the cuticle, and mechanism of action^5^. The larvicidal activity of EOs has been related to the presence of active compounds, such as terpenes and polypropanoids that might be neurotoxic or insect growth regulators^33^. Sesquiterpenes are reported to be more effective than monoterpenes, where the complex mixture might hamper the growth and survivability of insects. The *C.* *zeylanicum* essential oil (CZEO) displayed LC_90_ (49 ppm) and LC_50_ (37 ppm) values, respectively. The NE prepared using CZEO was observed to be spherical and exhibited 100% mortality within 3 days of treatment. The NE was stable even after dilution and exhibited upto 32% increased larvicidal activity compared to the EO^34^. A study with nanoformulations containing herbal oil and geraniol indicated a positive correlation between larvicidal activity and small droplet size^35^. It was also reported that *Azadirachta* *indica* NE was more effective against *Cu.* *quinquefasciatus* larvae^36^.

The larvicidal activity of Tarragon (*Artemisia* *dracunculus*) EO (TEO) exhibited an LC_50_ of 11.36 and LC_90_ of 17.54 ppm, respectively, against *An.* *stephensi* larvae. The comparative larvicidal activity of TEO and its NE F1 (2.5% ipa and Tw) and F4 (10%Tw) were observed to be 83, 82, and 93%, respectively. The significant increase in larvicidal properties from F1 to F4 might be because of the differences in the particle size^37^. The NE of *Rosmarinus* *officinalis* EO led to 80 ± 10 and 90 ± 10 mortality at 24 h and 48 h of exposure, respectively^8^. The nano-formulations of *B.* *reticularia* and D-limonene displayed IC_50_ values of 221.27 and 91.2534 µg/mL after 24 h, 144.68 and 81.95 µg/mL after 48 h of exposure against *A.* *aegypti* affecting larval development, mortality and damage to the digestive tube^38^. The NE formulation containing Basil oil, Tween-20, and water having a droplet size of 30 nm and PDI 0.234 caused 100% mortality of *A.* *aegypti* larvae at 100-fold dilution of the NE^39^. The larvicidal activity of Eucalyptus oil NE was more effective than its bulk counterpart against *Cu.* *quinquefasciatus.* The histopathological studies suggested damage to the plasma membrane, epithelial cells, and leakage of midgut contents^40^.

Temperature is an important factor which affects the insecticidal/larvicidal efficacy of EOs. Previous studies related to the EO based fumigation against pests^41,42^ and against *Pediculus* *humanus humanus* or *P.* *humanus capitis*^43^ have reported increased biological activities with an increase in temperature. There are relatively few studies with emphasis on post application temperature and the larvicidal properties of EOs. It has been reported that temperature causes significant changes in the mortality of *C.* *quinquefasciatus* larvae. At 10 °C lowest mortality (48%) was reported while at 20 °C highest mortality (80%) was observed. However, the mortality rates decreased with further rise in temperature^20^. Another study that evaluated the major compounds of *T.* *vulgaris* EO for acute toxicity against *C.* *quinquefasciatus* larvae reported similar results^44^. These studies lead us to the confirmation that the post-application temperature plays an important role in larvicidal efficacy of EOs. The results from these studies suggest that the temperature between 20 and 25 °C is optimal to obtain high mortality rates. In our temperature and humidity-controlled insectariums, we are routinely rearing and breeding *A.* *aegypti* and other mosquitoes which were maintained at 27 ± 1 °C and 75 ± 5% relative humidity (RH). However, we did not evaluated the effect of post application temperature on mortality and all the assays were performed at 27 °C. EOs and NE are considered very effective and eco-friendly however, only a few EO based botanical pesticides have been commercialized. One of the major reasons is the lack of studies on the effects of EOs on non test organisms. Most of the EOs and their components are reported to be not mutagenic or genotoxic in nature and the negative effects of ECs are observed only at high doses and also depend upon the exposure time. Several EOs have displayed toxicity below concentration limits set by international regulations. L(E)C50 values as low as 0.0336 mg L^−1^, 0.0005 mg L^−1^ and 0.0053 mg L^−1^ have been described for microalgae, crustaceans and fish, respectively^20,45,46^. EOs has also displayed low acute toxicity against Swiss albino mice, *Anisops* *bouvieri*, *Diplonychus* *indicus* and *Gambusia* *affinis*^20,47–50^. Muturi et al. evaluated the effect of 23 essential oils against three bacterial species (*B.* *cereus,* *Bacillus* *velezensis* *and* *Priestia* *megaterium*) that have been commercialized as bio stimulants and biocontrol antagonists. At a dilution of 1:10, seventeen of the 23 EOs displayed no inhibitory activity against any of the tested bacterial species^51^ mere characterization should not be presumed to equate with low toxicity.

Apart from the lethal effects of EO on target organisms several sublethal effects such as impairment of reproduction and feeding, impairment of behavioral traits, increased walking activity, histopathological changes in the midgut and repellent effects have also been observed^54^. The sublethal doses (LD30) of the Thyme oil when applied topically on the adults of the housefly (*Musca* *domestica* L.,) significantly decreased the longevity, fecundity and egg emergence. Only 50% of the larvae could emerge from the eggs laid by the treated female flies leading to more than 80% mortality rates^55^. The sublethal effects (fertility and potential natality) of carlina oxide found in *Carlina* *acaulis* EO were evaluated against adults of *Metopolophium* *dirhodum*. At sublethal doses of LC30 and LC50, carlina oxide inhibited the fertility by 35.68 ± 6.21% and 23.66 ± 10.58%, respectively while the potential natality of the target pest was inhibited by 52.78 ± 4.48% and 59.69 ± 5.60% respectively^56^. The EOs from *Bunium* *persicum* (Boiss.) *B.* *fedtsch* and *Ziziphorac* *linopodioides* Lam. and their 10% NEs displayed good efficacy against larvae and pupae of *Cu.* *quinquefasciatus*. The NEs of both the EOs arrested the larval development displaying significant sublethal toxicity as compared to the EOs^57^.

The physicochemical properties and insecticidal activity of *Origanum* *vulgare* (L.) (OV) and *Laurus* *nobilis* (L.) (LN) essential oils (EOs) in polymeric nanoparticles (PNs) were evaluated against the rice weevil (*Sitophilus* *oryzae* L.) and the cigarette beetle (*Lasioderma* *serricorne* F. OV PN significantly enhanced the lethal effect of EO against *S.* *oryzae* whereas both PNs were effective against *L.* *serricorne*. The PNs of OV and LN altered the nutritional physiology and behavior variables of both the tested pests and also prolonged the repellent effects of the EOs upto 60 and 48 h^58^. The sublethal effects are attributed to the presence of volatile or non-volatile substances in the EOs. Volatile substances can be attractive or repellent to insects, affecting the location of the host plant, oviposition and feeding of herbivorous insects. The non-volatile substances present in EOs can exert deterrence or arrestance effects on insects affecting the biological performance of insect pests. These sublethal effects, in synergy with the lethal effectscan lead to lower damage to the crop plants by the pests.

The appearance of insecticide resistance has been attributed to detoxifying enzymes such as non-specific esterases, glutathione S-transferases, and acetylcholinesterases. Non-specific esterase and acetylcholinesterases play vital functions in organophosphate and carbamate resistance, while glutathione S-transferases are an important enzyme associated with organochlorine and pyrethroid resistance^59^. Insects overcome the lethal effects of insecticides by the development of insecticide resistance mechanisms. Different types of resistance mechanisms have been observed, including target site resistance, and have been linked to acetylcholinesterase. The insecticides like organophosphates and carbamates target AChE, while pyrethroids and DDT inhibit voltage-gated sodium channels. The metabolic resistance is mainly related to increased expression or modified activity of the detoxifying enzymes such as esterases, mono-oxygenases, and glutathione-S-transferases (GST) that play a vital role in the detoxification of insecticides. Resistance occurs when increased levels or modified activities of enzymes prevent the insecticide from reaching its site of action. The metabolic and target site resistance mechanisms are found globally in different mosquito species. In India, resistance against DDT is extensive compared to pyrethroids or organophosphates^60,61^.

As per the literature, lower V_max_ and K_m_ values indicated enzyme activity that is activated below particular [S] and inhibited above it. In our assays, the presence of an inhibitor might have led to lower Km values due to high-affinity binding. In contrast, at higher substrate concentrations, saturation begins at the binding site, leading to a decrease in V_max_ values and causing enzymatic inhibition^62^. The low Vmax and raised Km values indicate that the α-NAE is inhibited by both the LCEO and LCNE as compared to the control. The lowered Vmax and lower Km values were observed in β-NAE as similar to the acetylcholine esterase, an indication of a similar mechanism of action of the LCEO or LCNE^62^. Lower Vmax and Lower Km observed for GST also indicate that the enzyme was activated below a particular substrate concentration and was inhibited at a substrate concentration above it^62^. It is to be noted that the increased expression levels of GST are observed in the resistant strains that might be degrading the insecticides at a higher rate. However, the inhibition observed by LCEO/LCNE might help reduce GST activity, thereby increasing effective insecticide concentrations.

The EO and NE exhibited inhibitory activities against all the tested enzymes. However, the LCNE was more potent than LCEO, displaying the importance of NE being used as carriers for insecticides or drugs. The inhibition of enzyme activity was mainly due to the complex chemical nature of the bulk oil that was enhanced by the use of highly soluble NE. The enzyme inhibition can be related to the anionic interaction of LCNE with the respective enzyme active site or at some other adjacent site. Green nanotechnology has opened new avenues for developing EO-based NE with larvicidal/ insecticidal activities. Very few studies have been carried out using NE as an insecticide and the effect of NE on enzyme kinetics. The results of our study demonstrate the efficacy of using NE as potent larvicides that can be utilized to tackle the emergence of insecticide resistance against synthetic insecticides.

## Conclusion

The LCEO was purified and used to prepare NE (O/W). The major components in the LCEO were Sabinene, β-cubebene, α-humelene, Germacrane-D and trans-Caryophyllenes. The current study demonstrated the green synthesis of environment-friendly NE’s that were stable in nature. The minimum particle size of the NE was 147.62 nm, with a PDI value of 15.1%. The LC_50_ values demonstrated that the LNCE (LC_50:_ 50.35 ppm) was potent larvicide compared to the EO (LC_50:_ 111.17 ppm). We observed that LCEO and LCNE inhibited acetylcholine esterase and α-non-specific esterase by noncompetitive inhibition, β-non-specific esterase by competitive inhibition, and Glutathione S-transferases by mixed inhibition. The inhibition of GST by LCEO/LCNE presents us with novel mechanisms to tackle the insecticide emergence against currently used insecticides. Further studies on the purified compounds present in the essential oil and their NE are required to pay the way for the development of environment-friendly insecticides against resistant mosquito strains.

## Materials and methods

### Plant collection and oil extraction

The *L.* *camara* leaves were collected in August 2017 around Indira Gandhi National Tribal University (IGNTU), Amarkantak, Madhya Pradesh, India. The identification of *L.* *camara* was certified by a subject expert (Dr. Ravindra Shukla, Associate Professor, Department of Botany, IGNTU) and a voucher specimen (SS/DOB/LC10) was deposited in the Department of Botany, IGNTU, Amarkantak, MP, India. The collection of the plant material and related studies complies with relevant institutional, national, and international guidelines and legislation. All methods presented in this manuscript were carried out in accordance with relevant guidelines, which are cited in the particular sections describing each method.

The collected leaves were washed thoroughly with distilled water to remove the dust particles. The oil was extracted in the Clevenger apparatus for about 7–8 h using the hydro-distillation method. The *L.* *camara* essential oil (LCEO) was collected, dried over anhydrous sodium sulfate, and stored at 4 °C until further analysis. Yellow-colored LCEO with a characteristic aroma was steam distilled with a yield of 0.3 mL/100 gm fresh leaves.

### GC/GC–MS analysis

#### GC analysis

The volatile components of LCEO were analyzed using a Perkin-Elmer Autosystem XL GC with Equity-5 capillary column of (60 mL × 0.32 mm internal diameter) and film 0.25 μm thickness. The carrier gas was Hydrogen at a flow of1mL/min. One µL aliquots of LCEO were manually injected in a split ratio 1:40. Oven programming was comprised of an initial temperature isotherm of 70 °C for 2 min, followed by a temperature ramp to 250 °C at 3 °C/min and a final isotherm at this temperature for 10 min. Injector and detector temperatures were set at 280 and 300 °C, respectively.

#### GC–MS analysis

Identification and characterization of the essential oil were carried out using Perkin Elmer Claus SQ 8 MS Clarus 680 GC with ELITE-5-MS (30 mL × 0.25 mm internal diameter × 0.25 μm thickness) gas column. The carrier gas was Helium at constant flow (1 mL/min). GC oven programming comprised of an initial temperature of 60–240 °C for (6 min) at 3 °C/min. The 0.02 µL of LCEO was injected at 250 °C by mass range 50–400 amu. The compounds were identified using a spectral database search (NIST and WILEY9 software library of mass spectra) and spectra reported in the literature^63^.

### Nanoemulsion formulation

The formation of LCNE was carried out using the low-energy approach that relies on the spontaneous formation of fine oil droplets within surfactant–oil–water (SOW) mixtures when their composition and/or environment are altered^64^. The oil-in-water (O/W) LCNE was formulated using water [75% (w/w)], oil [10% (w/w)], the surfactant Tween-20 (HIMEDIA-MB067) [10% (w/w)] and co-surfactant ethanol [5% (w/w)]. The preparation of LCNE was initiated by mixing oil and Tween-20 at 1200 rpm for 15 min using a magnetic stirrer at room temperature (RT). The absolute ethanol was added to the mixture for complete solubility and tight binding of oil and surfactant. The mixture was stirred for 5 min, followed by the addition of water in a drop-by-drop fashion at a flow rate of 3 mL/min^65^. This mixture was stirred at 1500 rpm for different durations, viz. 5, 10, 20, 30, and 60 min. The final products (LCNE) obtained after different time points were stored at RT. The LCNE was characterized by analysis of droplet size, size distribution, and stability using diluted NE.

### UV-Spectroscopy of LCNE

The LCNE was diluted (1:100) and analyzed using a UV–Visible spectroscope (Shimadzu 1800-UV–Vis spectrophotometer) between wavelength range 200–800 nm at different time intervals (5, 10, 20, 30, and 60 min). The peak values were recorded after baseline correction^66^. For analysis of the stability, the LCNE was centrifuged at 3000 rpm for 10 min and re-examined as mentioned above.

### Fourier Transform Infrared (FTIR) spectroscopic analysis of oil and NE

The FTIR analysis is the most potent tool for identifying the types of characteristic chemical bonds and functional groups in any essential oil and NE. The LCNE was diluted before scanning the FTIR spectrum at 400–4000 cm^−1^ (Thermo Scientific iD7 ATR Spectrophotometer), and the peak values were recorded. The analysis was performed in duplicates for confirmation of the obtained spectrum^67^.

### Droplet size and particle size distribution

Dynamic light scattering (DLS) method, also called photon correlation spectroscopy (PCS), analyzes the fluctuations in the intensity of scattering by particles due to Brownian motion^68^.Both droplet size and size distribution of LCNE were determined using Litesizer 500. To reduce the multiple scattering effects, the LCNE was 100 times diluted with water prior to use. Droplet size and size distribution were illustrated in nm and polydispersity index (PDI), respectively.

### Stability analysis of the nanoemulsion

#### Centrifugation

The formulated LCNE was centrifuged at 5000 rpm for 30 min and observed for phase separation, if any. Stable samples were further analyzed for heating and cooling cycles.

#### Heating–cooling cycle

The temperature stability analysis was carried out by keeping the formulated LCNE at 4 °C and 40 °C, alternating each temperature for 24 h. The cycle was repeated twice. The test helps to check the stability of LCNE at varying temperature ranges.

#### Zeta potential measurement

The zeta potential of the LCNE was carried out using Litesizer 500 at 25 °C. The sample was diluted 100 times in water before taking the measurement.

### Transmission electron microscopy

An emulsion was prepared in 1:10 dilution and sonicated in the ice-cold water bath for 1 min. 5µL of the sample was applied on the carbon-coated grid and was allowed to dry completely. The grid was stained with 1% phosphor tungstic acid (PTA) for 1 min. The excess stain was removed using blotting paper. The grid was left to be air-dried before being imaged using a transmission electron microscope (Thermo Fisher Scientific Talos F200) using a FEG filament operated at 200 kV.

### Scanning electron microscopy

The characterization of morphological features of the LCNE was imaged by a Scanning Electron Microscope (Carl Zeiss FESEM 03-81 Germany) operating at 5 kV capable of a point-to-point resolution. The sample was prepared by dropping a small amount of NE on the carbon-coated copper grid film. The grid was kept in a vacuum desiccator for overnight drying. The grid was placed inside the instrument, and the surface morphology was captured.

### Larvicidal and pupicidal activity

#### Rearing of mosquito larvae

Wild *An.* *culicifacies* mosquito larvae were collected from the IGNTU campus and transferred to enamel bowls containing water. Yeast tablets and dog biscuits purchased locally were crushed into fine powder separately in a mixer grinder. A 3:1 mixture of the powdered yeast tablets and dog biscuits was used as a feed for growing mosquito larvae maintained in an insectary at 27 ± 1 °C temperature and 75 ± 5% relative humidity (RH).The larvicidal and pupicidal activity of LCEO and LCNE were performed according to World Health Organization (WHO) protocol^69^. 1% stock solution was prepared (200µL of LCEO/LCNE in 19.8 mL of acetone/water). Further stock solution was diluted using water and prepared different dilutions (10, 50, 100, 200, 300, 400, and 500 ppm). Same concentrations of temephos were used as the positive control, and water was used as blank. In each beaker, 25 fourth instar larvae and 20 pupae were transferred individually. The experiment was conducted in triplicates. The mortality rate was observed after 24 h and at 48 h. The percent mortality was calculated using Abbott’s formula^69^ as follows:

$$\% {\text{of}}\;{\text{corrected}}\;{\text{mortality}} = \frac{{\% {\text{survival}}\;{\text{in}}\;{\text{untreated}}\;{\text{control}} - \% {\text{survival}}\;{\text{in}}\;{\text{treated}}\;{\text{sample}}}}{{\% {\text{survival}}\;{\text{in}}\;{\text{untreated}}\;{\text{control}}}} \times 100$$

### Biochemical analysis

#### Preparation of enzyme

The fresh, untreated mosquito larvae were used for inhibition assay. A total of 50 fourth instar larvae were weighted (90 mg) and homogenized in 2 mL of ice-cold 0.05 M phosphate buffer (pH 7.4) using a lab homogenizer (Remi) at 200 rpm^70^. The homogenate was diluted 3 times, adding phosphate buffer. As the acetylcholine esterase enzyme is membrane-bound, 3 mL of homogenate was separated before spinning. The remaining homogenate was centrifuged at 10000 rpm for 15 min at 4 °C^71^. The supernatant was used for Glutathione S-transferases and non-specific esterase inhibition activities. The protein concentration was measured using Bradford’s method^72^.

#### Acetylcholine esterase inhibition assay

The assay was carried out as per the WHO protocol with slight modifications^73,74^. Acetylcholine iodide (AChI; 0.01 M) and different inhibitor concentrations (20, 17.5, 10, 5, 3, 2.5, 2, 1, and 0.5 ppm) were prepared. To each well, 20 µL of homogenate was added, followed by 145 µL of Triton phosphate buffer (pH 7.8) for solubilizing the acetylcholine esterase. To this mixture, 10 µL of dithiobis-2-nitrobenzoic acid (DTNB) was added gently to the solution and mixed to avoid any bubbles. 25 µL of a mixture of AChI and inhibitor (1:1) was added to test wells. The control contained 20 µL of larval homogenate, 145 µL triton buffer, 10 µL of DTNB, and 25 µL of only AChI. The blank had only 40 µL of water, 145 µL of triton buffer, and 25 µL ofAChI. The absorption was read at 405 nm after 1 h as a fixed time point. The IC_50_ values were calculated based on the normalized response (%) and log [I]^75^.

The enzyme inhibition kinetic study was undertaken using the IC_50_ values that were obtained above. The substrate concentration was varied (1, 5, 10, 20, and 50 mM mL^−1^), and time-dependent absorbance values were recorded continuously for 5 min. The rate of reaction was calculated by using the extinction coefficient (ε = 13.6 mM^−1^ cm^−1^).

#### Non-specific esterase (NSE) inhibition assay

This assay measures esterase activity directly and uses two substrates [α and β naphthyl acetate (NA)]. The assay was performed as per WHO protocol with slight modifications^74^. 30 mM stock of α and β naphthyl acetate (α and β NA) was prepared in acetone. Inhibitors (LCEO/LCNE) at different concentrations (20, 17.5, 10, 5, 3, 2.5, 2, 1, and 0.5 ppm) were pre-incubated with homogenate for 30 min. The blank contained 20µL of distilled water, 200 µL of α NA or β NA, and the control wells contained 10 µL of homogenate and 10 µL of distilled water. In contrast, the test (LCEO/LCNE) well contained 20 µL pre-incubated homogenate mixture and 200 µL of α or β NA in all the wells. After adding NA, the plates were incubated for 15 min at RT. 50 µL of Fast blue B stain solution was added after the incubation (150 mg of fast blue salt in 15 mL of dist. Water + 35 mL of 5% SDS) to all wells of the plate. The absorbance was read at 570 nm after 15 min as a fixed time point. The IC_50_ values were calculated based on the normalized response (%) and log [I]. The enzyme inhibition kinetics was studied using the calculated IC50 values above. The substrate (α-NA and β-NA) concentration was varied (1, 5, 10, 20, and 50 mM/mL), and time-dependent absorbance values were recorded continuously for 15 min with 5 min intervals. The reaction rate was calculated as Umol/mL/min using α/β- naphthol standard calibration curve.

#### Glutathione S-transferases (GST) inhibition assay

The assay was carried out according to WHO protocol with some modifications^74^.The final 10 mM reduced glutathione (GSH) was prepared in 0.1 M phosphate buffer (pH 6.5). Chlorodinitrobenzene (CDNB) 63 mM/mL was prepared in methanol. The reaction mixture was prepared by adding 125 µL of CDNB and 2.5 mL of GSH. Reagents were prepared fresh prior to use. For blank, 20 µL of distilled water and 200 µL of the reaction mixture (CDNB + GSH) were added, while in the control assay, 10 µL of larval homogenate, 10 µL of distilled water, and 200 µL of the reaction mixture were added and mixed gently. For the test assay, 10 µL of larval homogenate, 10 µL of the test sample (LCNE/LCNO) in varying concentrations (20, 17.5, 10, 5, 3, 2.5, 2, 1, and 0.5 ppm) and 200 µL of the reaction mixture were added and mixed gently. The absorbance was read at 340 nm after 20 min of incubation. The IC_50_ values were calculated based on the normalized response (%) and log [I].

The enzyme inhibition kinetics were studied using the IC_50_ values obtained above. The substrate concentration was varied (1, 5, 10, 20, and 50 mM/mL), and time-dependent absorbance values were recorded continuously for 5 min. The GST activity was calculated in µmol CDNB conjugated/min/well using the extinction coefficient (ε = 5.3 mM^−1^ cm^−1^).

#### Data analysis

The percentage of mortality was calculated using Abbott’s formula, and log probit analysis was used to compute the LC_50_ and LC_90_ values. The Michaelis–Menten equation was used to calculate the Vmax and K_m_ values by plotting different substrate concentration v/s enzyme activity in GraphPad Prism 8.0.1(244) version. The non-covalent inhibitor potency of enzymes was studied by determining their IC_50_ values (concentration of inhibitor to block half of the enzyme activity). A lower IC_50_ value shows a better potency of inhibition. Dose–response curves were plotted using absorbance (%) against the logarithmic concentrations of the inhibitors using GraphPad Prism 8.0.1(244) version.

### Supplementary Information

Supplementary Information.

## Data Availability

The data can be accessed/shared with the public.
