# Supplementary material for: In vitro larvicidal efficacy of Lantana camara essential oil and its nanoemulsion and enzyme inhibition kinetics against Anopheles culicifacies
Source: Sci Rep. 2024 Jul 15;14:16325. doi: 10.1038/s41598-024-67148-w (PMC11250815; doi:10.1038/s41598-024-67148-w)
Supplement: Supplementary file 1 — Supplementary Information. [file 41598_2024_67148_MOESM1_ESM.docx]

***In vitro* larvicidal efficacy of *Lantana camara* essential oil and its nanoemulsion and enzyme inhibition kinetics against *Anopheles culicifacies***

Shruti Sonter^a^, Manish Kumar Dwivedi^a,b^, Shringika Mishra**^a^**, Prabhakar Singh^c,^, Ramesh Kumar^e^, Sungmin Park^d*^, Byong-Hun Jeon^e,^***** and Prashant Kumar Singh^a,f^*****

^a^Department of Biotechnology, Indira Gandhi National Tribal University, Amarkantak (MP), India

^b^Hikal Limited, R&D, Hinjawadi, Pune, Maharashtra, India

^c^Sophisticated Analytical Instrumentation Facility, Department of Anatomy,

All India Institute of Medical Sciences, New Delhi

^d^Department of Civil and Environmental Engineering, Hanyang University, 222-Wangsimni-ro, Seongdong-gu, Seoul 04763, Republic of Korea

^e^Department of Earth Resources and Environmental Engineering, Hanyang University, Seoul, Republic of Korea

^f^Department of Biochemistry, University of Lucknow, Lucknow, Uttar Pradesh, India

*Email: prashantcdri@gmail.com

Phone No.: +91-9179122557

Shruti Sonter (former affiliation)

Department of Biotechnology, Indira Gandhi National Tribal University,

Amarkantak- 484887, Madhya Pradesh, India.

E-mail: [shrutiasonter07@gmail.com](mailto:shrutiasonter07@gmail.com)

Manish Kumar Dwivedi

Hikal Limited, R&D, Hinjawadi, Pune, Maharashtra, India

E-mail: [dwivedi.manish55@gmail.com](mailto:dwivedi.manish55@gmail.com)

Shringika Mishra (former affiliation)

Department of Biotechnology, Indira Gandhi National Tribal University,

Amarkantak- 484887, Madhya Pradesh, India.

E-mail: [shringik](mailto:shringika99@gmail.com)[a99@gmail.com](mailto:a99@gmail.com)

Sungmin Park

Department of Civil and Environmental Engineering, Hanyang University, 222-Wangsimni-ro, Seongdong-gu, Seoul 04763, Korea

E-mail: smpark@gns-eng.com

Ramesh Kumar,

Department of Earth Resources & Environmental Engineering, Hanyang University, 222-Wangsimni-ro, Seongdong-gu, Seoul 04763, Republic of Korea

E-mail: rameshibt@hanyang.ac.kr

Prabhakar Singh

Associate Professor

Sophisticated Analytical Instrumentation Facility, Department of Anatomy,

All India Institute of Medical Sciences, New Delhi, India

E-mail: [prabhakar.singh@aiims.edu](mailto:prabhakar.singh@aiims.edu)

Byong-Hun Jeon

Department of Earth Resources & Environmental Engineering, Hanyang University, 222-Wangsimni-ro, Seongdong-gu, Seoul 04763, Republic of Korea

bhjeon@hanyang.ac.kr

*Joint corresponding author: Prashant Kumar Singh, PhD, Sungmin Park, Byong-Hun Jeon, PhD

Tel: +91-9179122557

E-mail: prashantcdri@gmail.com,

ORCID ID: 0000-0002-6704-277X

Table S1: Larvicidal activity of *L. camara* oil and Nanoemulsion

| **Concentration in ppm** | **Percent mortality after 24 hrs**  **(Mean±SD)** | | | **Percent mortality after 48 hrs**  **(Mean±SD)** | | |
| --- | --- | --- | --- | --- | --- | --- |
|  | **Temephos** | **LCEO** | **LCNE** | **Temephos** | **LCEO** | **LCNE** |
| 10 | 9.4±0.57 | 5.4±0.5 | 8.5±1.5 | 16.1±0.5 | 9.4±1.5 | 15.9±1.5 |
| 50 | 17.5±1.15 | 13.5±0.5 | 28.5±1.5^**^ | 41.8±1.1 | 28.3±2.0 | 57.9±0.5^*^ |
| 100 | 24.3±1.52 | 20.2±0.5 | 48.5±1.0^**^ | 60.8±1.5 | 47.3±1.0 | 73.9±1.0 |
| 200 | 60.8±2.08 | 44.6±0.5^*^ | 68.5±0.5 | 82.4±1.5 | 59.4±1.0^**^ | 85.5±0.5 |
| 300 | 82.4±0.57 | 71.6±1.0^**^ | 88.5±0.5 | 95.9±1.0 | 75.6±1.0^**^ | 98.5±0.5 |
| 400 | 98.6±0.57 | 93.2±1.5 | 98.5±0.5 | 100±0.5 | 95.9±1.0 | 100±00 |
| 500 | 100±0.0^#^ | 100±0.0^#^ | 100±00^#^ | 100±0.0^#^ | 100±0.0^#^ | 100±0.0^#^ |
| **Control** | 0.01±0.57 | 0.01±0.5 | 0.6±0.57 | 0.01±0.5 | 0.0±0.57 | 0.6±1.0 |

^#^100% mortality in 30mins, p value <0.033 was considered significant and marked as *, p <0.002 as highly significant and marked as **, p <0.001 was very highly significant and marked as ***.

Table S2: Pupicidal activity of *L. camara* oil and Nanoemulsion

| **Concentration in ppm** | | **Percent mortality after 24 hrs**  **(Mean±SD)** | | | **Percent mortality after 48 hrs**  **(Mean±SD)** | | | |
| --- | --- | --- | --- | --- | --- | --- | --- | --- |
|  |  | **Temephos** | **LCEO** | **LCNE** | **Temephos** | **LCEO** | | **LCNE** |
| 10 | 5.1±0.5 | | 1.6±0.5 | 10.1±1.5 | 17.2±0.5 | 10.3±0.5 | 31.0±1.5^*^ | |
| 50 | 20.3±1.5 | | 13.5±0.0 | 40.6±0.5^**^ | 22.4±0.5 | 25.8±0.5 | 56.8±0.5^***^ | |
| 100 | 47.4±0.5 | | 28.7±1.0^***^ | 61.0±0.5^**^ | 58.6±0.5 | 43.0±1.0^**^ | 86.2±0.5^***^ | |
| 200 | 71.1±0.5 | | 55.9±0.5^**^ | 83.0±0.5^*^ | 84.4±0.5 | 65.5±0.5^**^ | 96.5±0.5^**^ | |
| 300 | 83.0±0.5 | | 77.9±0.5 | 94.9±1.0^*^ | 100±00^#^ | 93.1±0.5 | 100±00^#^ | |
| 400 | 94.9±0.0 | | 100±00^***/#^ | 100±00^***/#^ | 100±00^#^ | 100±00^#^ | 100±00^#^ | |
| 500 | 100±00^#^ | | 100±00^#^ | 100±00^#^ | 100±00^#^ | 100±00^#^ | 100±00^#^ | |
| **Control** | 0.01±0.5 | | 0.03±0.5 | 0.03±0.5 | 1.7±0.5 | 0.01±0.5 | 0.01±0.0 | |

^#^100% mortality in 30mins,p value <0.033 was considered significant and marked as *, p <0.002 as highly significant and marked as **, p <0.001 was very highly significant and marked as ***.

**Figures:**

**
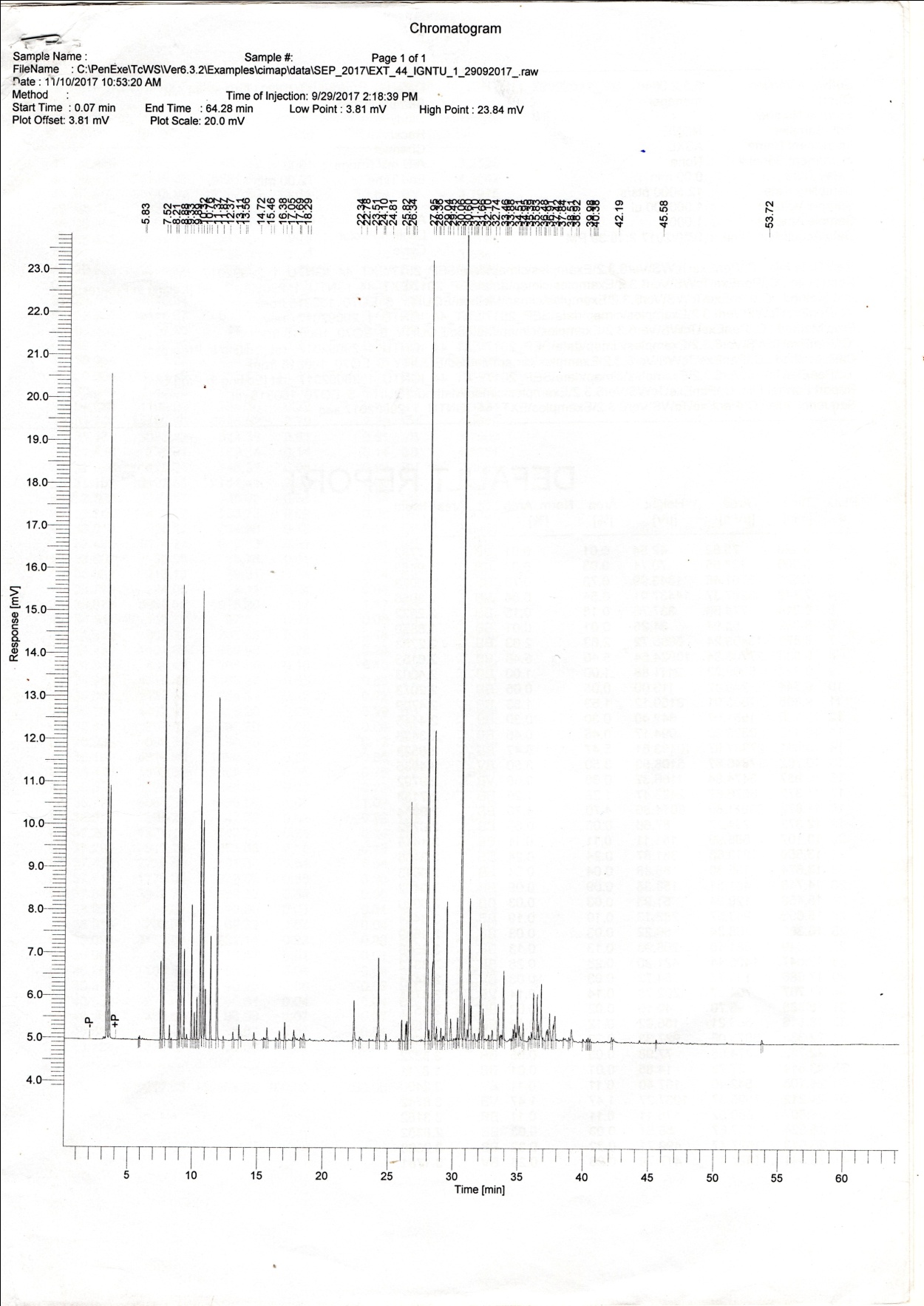
**

**Figure S1: Gas chromatogram of *L. camara* essential oil**


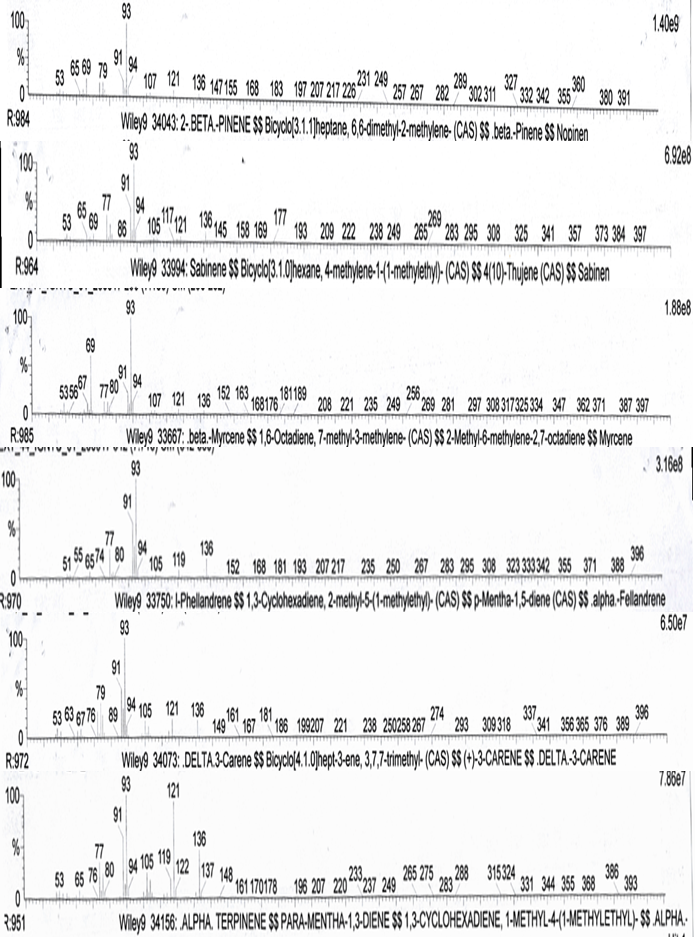


**Figure S2: Major compounds identified through GC-MS of *L. camara* essential oil**


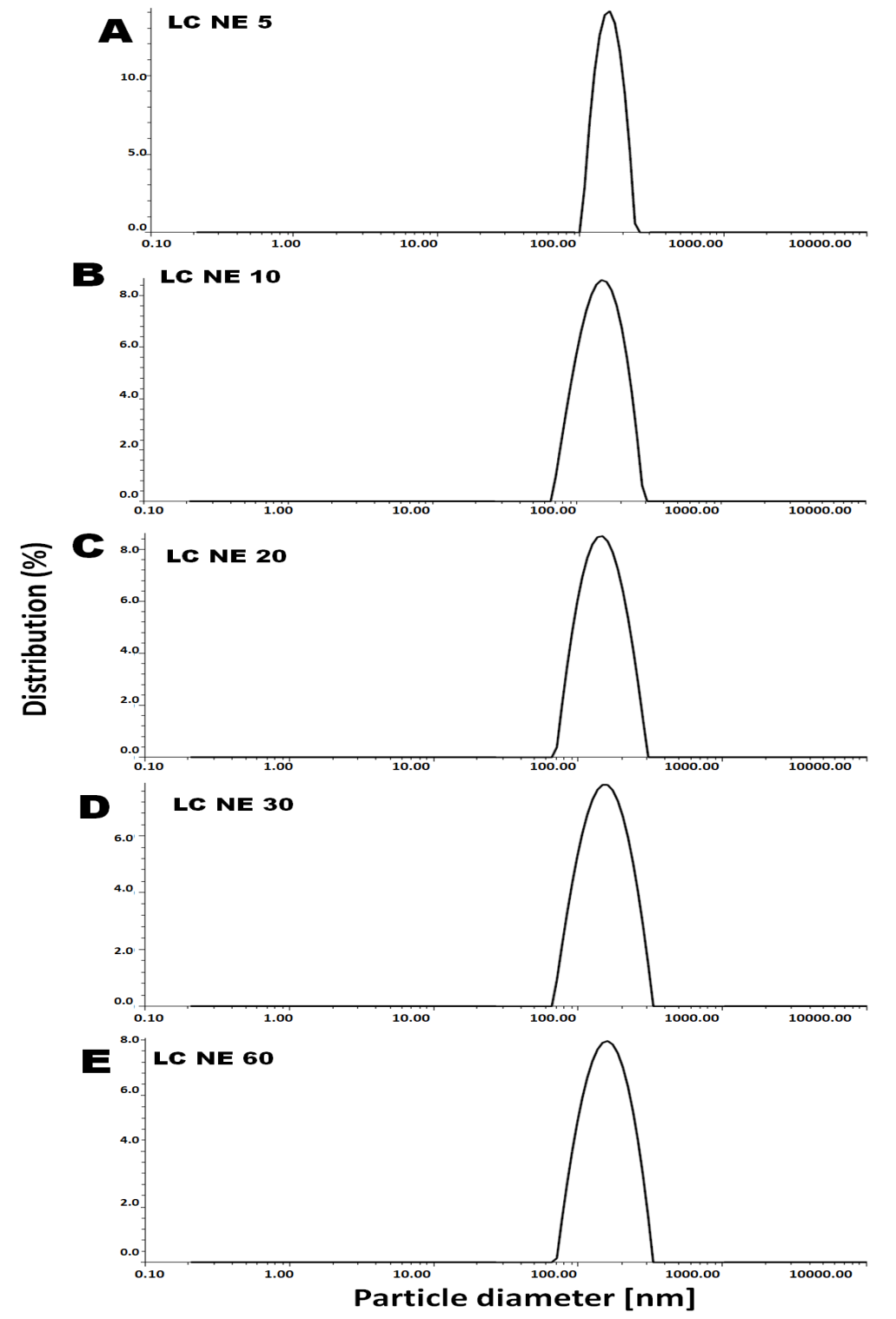


**Figure S3: Particle size analyze of *L. camara* Nanoemulsion (LCNE) at different time intervals. A: LCNE 05, B: LCNE-10, C: LCNE-20, D: LCNE-30, E; LCNE- 60**


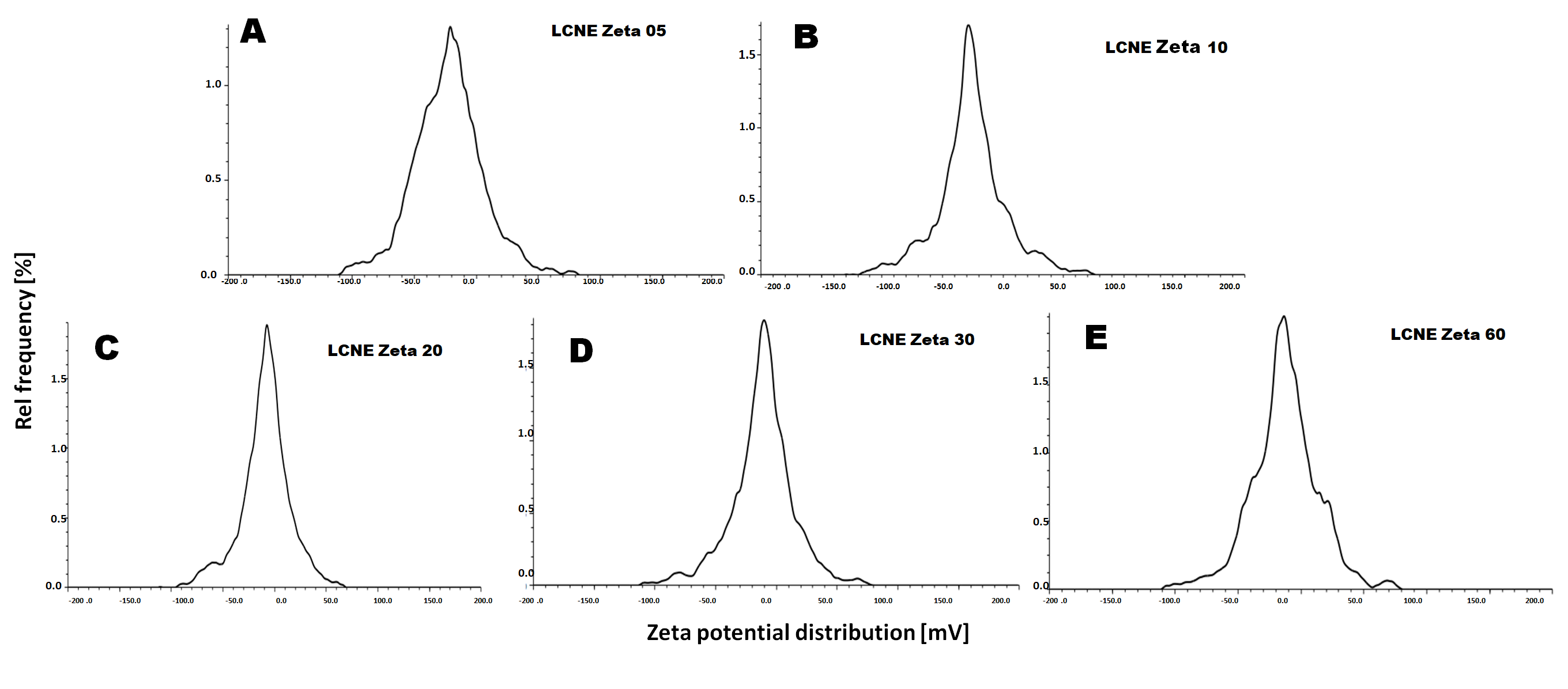


**Figure S4**. **Zeta potential of *L. camara* Nanoemulsion (LCNE) at different time intervals. A: LCNE 05, B: LCNE10, C: LCNE20, D: LCNE30, E; LCNE60**
